# Pancreatic Adenocarcinoma Therapeutics Targeting RTK and TGF Beta Receptor

**DOI:** 10.3390/ijms22158125

**Published:** 2021-07-29

**Authors:** Hsin-Han Yang, Jen-Wei Liu, Jui-Hao Lee, Horng-Jyh Harn, Tzyy-Wen Chiou

**Affiliations:** 1 Department of Life Science and Graduate Institute of Biotechnology, National Dong Hwa University, Hualien 974, Taiwan; 810413102@gms.ndhu.edu.tw; 2 Everfront Biotech Inc., New Taipei City 221, Taiwan; coldwee@efbiotech.com (J.-W.L.); juihaolee@efbiotech.com (J.-H.L.); 3 Bioinnovation Center, Tzu Chi Foundation, Buddhist Tzu Chi General Hospital, Tzu Chi University, Hualien 970, Taiwan; 4 Department of Pathology, Buddhist Tzu Chi General Hospital, Tzu Chi University, Hualien 970, Taiwan

**Keywords:** receptor tyrosine kinases, TGF beta receptor, pancreatic ductal adenocarcinoma, cancer- associated fibroblasts

## Abstract

Despite the improved overall survival rates in most cancers, pancreatic cancer remains one of the deadliest cancers in this decade. The rigid microenvironment, which majorly comprises cancer-associated fibroblasts (CAFs), plays an important role in the obstruction of pancreatic cancer therapy. To overcome this predicament, the signaling of receptor tyrosine kinases (RTKs) and TGF beta receptor (TGFβR) in both pancreatic cancer cell and supporting CAF should be considered as the therapeutic target. The activation of receptors has been reported to be aberrant to cell cycle regulation, and signal transduction pathways, such as growth-factor induced proliferation, and can also influence the apoptotic sensitivity of tumor cells. In this article, the regulation of RTKs/TGFβR between pancreatic ductal adenocarcinoma (PDAC) and CAFs, as well as the RTKs/TGFβR inhibitor-based clinical trials on pancreatic cancer are reviewed.

## 1. Introduction

**Figure 1:**
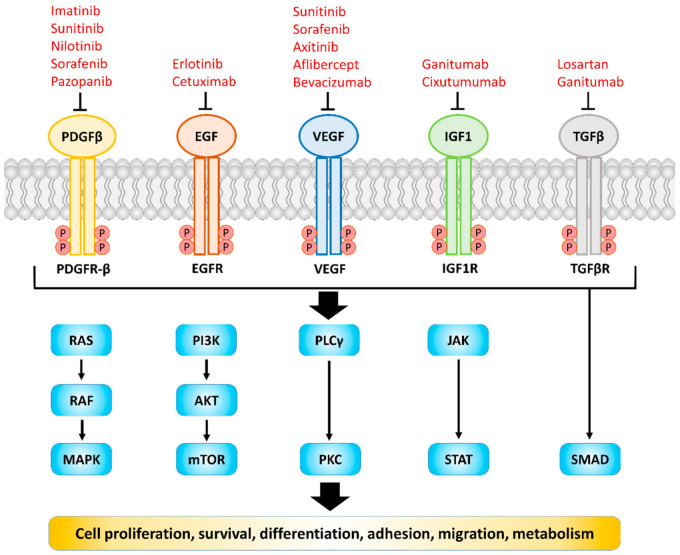
RTKs/TGFβR and their ligands which involved in regulating biochemical signal and cellular behaviors. Dysregulation of these receptors and ligands is found in most human cancers. Representative drugs targeting corresponding RTKs/TGFβR are shown. RAS, rat sarcoma; RAF, rapidly accelerated fibrosarcoma; MAPK, mitogen-activated protein kinase; PI3K, phosphatidylinositol-3-kinase; AKT, protein kinase B; mTOR, mechanistic target of rapamycin; PLCγ, phospholipase C-γ; PKC, protein kinase C; JAK, Janus kinase; STAT, signal transducer and activator of transcription.

Receptor tyrosine kinases (RTKs) and TGF beta receptor (TGFβR) are transmembrane proteins expressed on the cell membrane, the structure of which includes the ligand binding domain, the transmembrane helix outside the cell membrane, and the area containing message regulation, tyrosine/serine/threonine kinase domain and C terminal tail [1,2]. The substrates of RTKs and TGFβR can be provided by the cell autocrine manner or the paracrine/juxtacrine manner from the surrounding cells. Once bound to the corresponding substrate, the tyrosine kinase domain (in the case of RTKs) and serine/threonine domain (in the case of TGFbR) will be activated, and initiate the downstream signaling axis, ultimately regulating physiological responses such as cell growth, morphology, and metabolism [1,2] (Figure 1). Since they are highly sensitive and have characteristics of initiating signal cascades, the regulation of these receptors is tightly controlled. Once the expression or the secretion of their corresponding substrates is dysregulated, many diseases, especially cancer, will occur [3,4]. The correlation between dysregulated RTKs/TGFβR signaling and poor overall survival in patients has been demonstrated in many studies [5,6,7,8,9,10,11]. Hence, they have become one of the important targets in clinics. Nowadays there are numerous antibodies or inhibitors against RTKs/TGFβR including erlotinib, regorafenib and bevacizumab, which have therapeutic effects on many cancers such as non-small cell lung cancer (NSCLC) and colorectal cancer [12,13]. However, most of them failed to exert clinical benefits on patients with pancreatic ductal adenocarcinoma (PDAC) (Table 1). Since the extensive and rigid desmoplastic stroma, which accounts for up to 90% of the tumor microenvironment (TME), has been demonstrated to play a crucial role in enhancing the proliferative, invasive and immunosuppressive properties of PDAC [14], the further understanding of the effect of RTKs/TGFβR on TME composed cells, especially CAFs, are needed. Therefore, this article will review various RTKs/TGFβR and the related signaling axis on the cancer cells as well as CAFs within the TME.

## 2. The Clinical Significance of RTKs in PDAC Tumor Microenvironment and Related Trials

### 2.1. Platelet-Derived Growth Factor Receptors (PDGFRs)

The platelet-derived growth factors (PDGFs) and their receptors (PDGFRs) are crucial regulators in the vascular development and embryonic organogenesis, such as the cardiac neural crest, lung, intestine, skin, CNS, and skeleton. PDGF isoforms are often expressed by epithelial cells, whereas PDGFRs, including PDGFRα and PDGFRβ, are mostly expressed in cells of mesenchymal origin, such as fibroblasts, VSMCs and pericytes [28]. PDGFRs control the functions of these cells through remodeling the actin cytoskeleton, promoting cell growth by activating the RAS-MAPK and Src pathways, and inducing cell migration by activating PI3K-AKT and PLC-γ [29]. In addition, the cells of mesenchymal origin also produce ECM components including collagen, hyaluronan, laminin, which is required for integrin-mediated and focal adhesion formation of cancer cells to activate intracellular signaling pathways and downstream behavioral effects such as migration, invasion, and survival during the crosstalk of mesenchymal origin cells and cancer cells [30,31]. Given these properties, the recruiting and the fibrogenesis of CAFs in malignancies, such as breast [32], lung [33] and colorectal cancers [34] are thought to reflect paracrine effects of tumor cell-secreted PDGF. Besides, Cullen et al. have demonstrated that PDGF secreted by cancer cells induces CAFs proliferation and IGF1/2 expression. Notably, the CAFs-secreted IGFs in turn trigger the proliferation of cancer cells and the synthesis of PDGF [35], resulting in a positive feedback loop which exacerbates the tumor progression.

In addition to the role of PDGFRs in cancer progression, they may serve as markers to subtype CAFs, in which these CAFs subtypes may differentially affect the tumor microenvironment and clinical prognosis. For example, in preclinical study, PDGFRα has been reported to highly express on matrix CAF (mCAF) at the invasive front of breast tumors, compared to the tumor core, at which the abundance of mCAF are relatively low [36]. In another study, Costa et al. demonstrated that PDGFRβ highly expressed CAF-S1 subtype from four classified CAFs resected human breast tumor samples can attract and retain Treg cells through ligand for OX40 (OX40L), programmed cell death-ligand 2 (PD-L2), and junctional adhesion molecule 2 (JAM2), which contributed to an immunosuppressive microenvironment [37]. In clinical settings such as breast cancer and NSCLC, the increased PDGF expression in cancer cells, and PDGFRβ expression in CAFs are positively correlated with poor prognosis and short overall survival (OS) [8,38]. For PDAC, Yuzawa et al. have provided the clinical evidence that patients who are strongly PDGFRβ positive on the tumor stroma are significantly worse off in terms of lymphatic invasion, lymph node metastasis and median OS (22.5 months for lower PDGFRβ vs. 13.0 months for higher PDGFRβ) than those with intermediate or weak positivity [39,40,41]. Although typically seen alongside with other biomarkers, representing dynamic and interconvertible states in different microenvironments, most classified CAFs from PDAC, including inflammatory CAF (iCAF), myofibroblasts CAF (myCAF), antigen-presenting CAFs (apCAF), Sub-type A to Sub-type D CAF, share the expression of PDFGRα or PDFGRβ [42,43]. Hence, targeting to PDGFRs to inhibit the differentiation of normal mesenchymal-originated cells into those CAFs, and their positive interacting loop with PDAC, as mentioned earlier, becomes a potential therapeutic strategy.

Several potent PDGFRβ inhibitors, such as sunitinib, imatinib, nilotinib and sorafenib, have been developed for treating malignancies including chronic myelogenous leukemia (CML) [44], renal cell carcinoma (RCC) [45] and hepatocellular carcinoma (HCC) [46]. However, these inhibitors are multitargeted to other kinases and lack specificity. Sunitinib, for example, also inhibits vascular endothelial cell growth factor (VEGF) receptor, and has been tested in PDAC maintenance therapy [16]. In this study, the progression free survival (PFS) significantly improved in the sunitinib group compared to the observation alone group (*p* < 0.01; hazard ratio (HR) 0.51; 95% CI 0.29–0.89) [16]. However, in another phase II clinical study, the OS of PDAC patients did not reach significant improvement in the sunitinib group or the group receiving sunitinib with gemcitabine [17].

Imatinib is also a multikinase inhibitor targeting stem cell receptor (c-Kit) and Abl kinases. In a Phase II trial, it was used as a first-line and single-agent therapy in unresectable metastatic PDAC patients. However, patients who orally received 400 mg imatinib twice a day for a 28-day period did not have an improved OS in this study, so the patients did not remain on treatment for 3 months and the median OS was 118 days (ranged from 40 to 221 days). In the other phase II single-arm study, the group who took imatinib in combination with standard gemcitabine therapy did not show better PFS or OS benefits compared to those taking gemcitabine alone [15].

Sorafenib is a multiple tyrosine kinase inhibitor of PDGFRs, VEGF receptor (VEGFR) and serine/threonine-protein kinase B-raf (BRAF). In the phase II and phase III clinical trial, the groups of patients with advanced or metastatic PDAC received gemcitabine in combination with or without sorafenib. However, both studies showed no clinical benefit in PFS or OS in the treatment of these advanced PDAC patients [47].

Taken together, targeting the PDGFR alone, either alpha form or beta form, did not show therapeutic efficacy in patients with pancreatic cancer. Although these inhibitors can target multiple RTKS in the oncogenic process, they produced limited benefit, with only one trial showing PFS improvement, but not OS. The limitation in common, as mentioned by Moss et al., is that the current clinical studies did not analyze patient tumor specimens [15]. Besides, Gonçalves et al. have illustrated that other pathways may be upregulated to circumvent specific RTK inhibition, particularly in PDAC, which can only be observed by histopathological examination [21]. Hence, evaluating clinical activity in patients for addressing the RTK expression status, and answering whether this expression correlates with drug responses, may provide perspectives for developing novel drugs or therapeutic strategies future.

### 2.2. Epidermal Growth Factor Receptor (EGFR)

The epidermal growth factor receptor (EGFR) is one of the ErbB family members which expresses on almost all cell types except for hematopoietic cells [48,49]. The major signaling pathways activated by EGFR are the RAS-MAPK, PI3K-AKT-mTOR and PLC-γ1-PKC pathways, which lead to the cell cycle progression and exert critical functions such as differentiation, proliferation and survival in epithelial cell physiology as well as normal embryogenesis of vertebrates [50,51]. It has been reported that the tumor progression and invasion are driven by the ECM-dependent mechanosensitization of EGFR signaling [52,53]. The increased ECM stiffness promotes the expression of focal adhesion kinase (FAK) and PI3K, and favors EGFR-dependent squamous cell carcinoma (SCC) proliferation and invasion, which is orchestrated by CAFs [53,54]. For PDAC, Hu et al. showed that the elevated level of COX-2 was activated by EGFR/p38-MAPK/Sp1 signaling axis and led to the angiogenesis [55]; Ma et al. has demonstrated that the transformation of macrophage from M1 to M2 is triggered by pancreatic cancer secreted regenerating family member 4 (REG4) through the EGFR/AKT/CREB (cAMP-response element binding protein) pathway [56].

Driven largely by these roles, dysregulated EGFR has been vilified as a proto-oncogene by several oncogenic mechanisms, including overexpression as a consequence of EGFR gene mutation, gene amplification, and EGFR protein overexpression found in several types of tumors especially NSCLC [57,58]. In NSCLC, the short deletions in exon 19 and the G719S, L858R, and L861Q point mutation in exon 21 account for around 90% of all EGFR gene mutations, and therefore they predict better responsiveness to anti-EGFR therapy [58,59,60]. Given that the antibodies and small molecule inhibitors against EGFR exerted therapeutic effects on NSCLC patients, therapeutic strategies of EGFR inhibition may enable conceivable benefits in PDAC treatment, and have been developed and tested in pre-clinical and clinical studies.

In a phase III trial, a monoclonal anti-EGFR antibody cetuximab was previously used in locally advanced or metastatic PDAC patients. However, among the 745 eligible patients recruited, no significant difference was observed between the group of gemcitabine plus cetuximab and gemcitabine alone. The median OS and PFS of gemcitabine with cetuximab treatment were 6.3 and 3.4 months, respectively, compared to 5.9 and 3.0 with the administration of gemcitabine alone (OS: *p* = 0.19; hazard ratio 1.06; 95% CI 0.91 to 1.23; PFS *p* = 0.18; hazard ratio 1.07; 95% CI 0.93 to 1.24). In addition, the objective response rates were low in both groups (*p* = 0.59). The partial (or unconfirmed) response in gemcitabine alone group and combination group was 14% and 12%, respectively, whereas the stable disease was 30% and 37% [19]. In contrast to protein drug treatment, Moore et al. showed that the erlotinib, a small molecule RTK inhibitor, provided therapeutic benefit on advanced PDAC [18]. Compared to the group of standard gemcitabine in combination with placebo, patients received with standard gemcitabine plus erlotinib (100 or 150 mg/d orally) exhibited improved OS (*p* = 0.038; hazard ratio 0.82; 95% CI 0.69 to 0.99) and PFS (*p* = 0.04; hazard ratio 0.77; 95% CI 0.64 to 0.92). Besides, a randomized, open-label, prospective trial, the gemcitabine plus erlotinib group revealed that disease control (85% vs. 33%; *p* = 0.001), progression-free survival (median 5.9 vs. 2.4 months; *p* = 0.004), and overall survival (median 8.7 vs. 6.0 months; *p* = 0.044) were better in patients with EGFR mutations than in those without EGFR mutations [61]. Although the EGFR and K-RAS mutations are mutually exclusive in clinical observations of NSCLC and colorectal cancer, they have been found to co-exist in PDAC despite EGFR mutation only accounting for less than 3% of PDAC [62,63]. Furthermore, the EGFR signaling is required to initiate the KRAS oncogene-driven PanIN lesions and PDAC [63], and may be the possible reason that erlotinib exerts therapeutic effects for patients with pancreatic cancer. Besides, small molecule drugs with lower molecular sizes than protein drugs could penetrate the complicated stromal environment to modulate CAFs and inhibit cancer cell proliferation [64]. Moreover, erlotinib possesses multiple kinase targeting capabilities that could be used to expand benefits for patients with cancers [65]. Having an understanding of specific genetic mutations in patients with pancreatic cancer may be crucial and critical for customized medicine. Although the EGFR inhibitor erlotinib has been given the green light from the FDA to treat pancreatic cancer for over 10-year period, detailed downstream signaling targets, e.g., KRAS [66], are promising and require further study.

### 2.3. Vascular Endothelial Growth Factor Receptor (VEGFR)

The VEGFR is well known for its role in angiogenesis, which is favored for the metastasis of many cancers. However, PDAC is characterized by a low microvascular density and hypoxia with low drug delivery compared to other types of malignancies, despite the fact that VEGFR is overexpressed in over 90% of patients with PDAC [67]. Due to the characteristics of its vascular collapse, strategies targeting VEGFR seem to be ineffective. In a phase III trial, Kindler et al. investigated the therapeutic effect of the gemcitabine alone and the combination of gemcitabine with bevacizumab, which can neutralize VEGF to inhibit its activation. Nevertheless, among the 535 eligible patients recruited, the OS and PFS did not improve in the group receiving bevacizumab therapy [20]. The median OS for gemcitabine plus bevacizumab or placebo were 5.9 and 5.8 months, respectively (*p* = 0.95; hazard ratio 1.44; 95% CI 0.88 to 1.24). The median PFS for the gemcitabine with or without bevacizumab groups were 3.8 and 2.9 months, respectively [20]. Moreover, the phase II study showed that the combination of bevacizumab with docetaxel did not show the benefit in gemcitabine-refractory metastatic PDAC [68]. In addition, another phase III trial evaluating the therapeutic effect of bevacizumab in combination with gemcitabine plus erlotinib was conducted. Although the PFS was significantly improved (4.6 months when treated with gemcitabine-erlotinib plus placebo vs. 3.6 months when treated with gemcitabine-erlotinib plus bevacizumab) in the enrolled patients without severe side effects (*p* = 0.002; hazard ratio 0.73; 95% CI 0.61 to 0.86), the OS (6.0 months of gemcitabine-erlotinib plus placebo vs. 7.7 months of gemcitabine-erlotinib plus bevacizumab) did not significantly extend life expectancy in the bevacizumab addition group (*p* = 0.2087; hazard ratio 0.89; 95% CI 0.74 to 1.07) [69]. Combination therapies using other anti-angiogenic agents, such as sunitinib [17], sorafenib [21], axitinib [22], and ZIV-aflibercept [23], in addition to gemcitabine in phase III trials have also yielded negative results. These reports have suggested that the specific therapy targeting only VEGF will not enter clinical practice. Hence, additional studies inhibiting multiple targets in PDAC are still needed to lead to more encouraging results. The multikinase inhibitor Regorafenib has been reported to exert antiangiogenic activity. In an ongoing phase II trial (NCT02080260), regorafenib is being administered in metastatic PDAC patients after chemotherapy with gemcitabine. In addition, a phase II study of Ramucirumab (NCT02307500), a human monoclonal antibody against human VEGFR-2, is also ongoing to evaluate the efficacy and safety of combining it with the FOLFIRINOX regimen in 94 subjects with advanced progression.

### 2.4. Insulinlike Growth Factor 1 Receptor (IGF1R)

IGF1R is an insulin receptor family member which is often expressed in low tissue specificity, serving multiple physiological functions in cell growth and embryonic development [70]. The importance of IGF1R in regulating the differentiation of muscle [71], as well as maintaining the myocardium and brain, has been demonstrated [72]. Furthermore, IGF1R contributes to glucose metabolism and the physiology of neutrophils [73], and is associated with the development of diabetes, inflammation and cardiovascular disorder [74,75]. Within the PDAC tissues and correlated with higher tumor grades, it has commonly been found that IGF1R is highly expressed and co-expressed with EGFR, this is likely to lead to poor survival [76].

It has been demonstrated that the sensitivity of the tumor to hypoxia, low pH and blood glucose concentration can be reduced by IGF1R [77], suggesting the potential roles of IGF1R signaling in the development the hypoxic TME of PDAC. Apart from studies demonstrating the roles of IGF signaling on cancer cells in PDAC, the impact of stromal derived IGFs has been reported recently. In clinics, more than 90% of PDAC cases are due to the mutation of KRAS, which have been reported to activate fibroblasts through the Sonic Hedgehog (Shh) pathway [78]. The activation of the fibroblast initiates the positive feedback loop, which secretes IGF1 and further activates IGF1R on cancer cells [78,79,80]. Hence, the activated stromal (myo-) fibroblasts may be the foremost source of IGF1.

Therapeutic strategies targeting IGF1R or IGF ligands have been developed and tested in pre-clinical and phase I/II clinical studies. However, none of advanced clinical trials showed an improved clinical outcome in the combination of IGF1R or EGFR inhibitor with gemcitabine, compared to the patients that received gemcitabine monotherapy in advanced clinical trials (NCT01231347, NCT00819169, NCT00617708) [24,25]. As the CAFs have also recently been identified as one of the sources secreting IGFs, the roles of CAFs in regulating the IGF signaling axis in the TME of PDAC need to be further studied to develop novel therapeutic strategies of dual targeting on stroma and cancer cells.

## 3. The Clinical Significance of Transforming Growth Factor Beta Receptor (TGFβR) in PDAC Tumor Microenvironment and Related Trials

TGFβR is a serine/threonine kinase that plays a key role in regulating tissue homeostasis, cellular adhesion, differentiation, proliferation and survival. In normal tissues, TGFβ signaling leaves the epithelial cells in a quiescent state with a low proliferation rate, which reflects its tumor-suppressor activity [81,82]. In the circumstances of injury, the epithelial tissue is repaired by the recruited cells such as α-SMA positive myofibroblasts, M2 macrophages, and newly formed blood vessels as well as their secreted ECM. As with the above process, tumor-derived TGFβ is likely to recruit stromal cell types characterized by CAFs. In the established cancers, the roles of TGFβ on oncogenesis, such as promoting cancer cell proliferation, epithelial-to-mesenchymal transition (EMT), invasion, and metastasis, have been well evidenced [83,84,85]. For PDAC, the enhanced expression of TGFβ and TGFβR is associated with advanced tumor stage and lower OS [86,87,88].

The importance and dependence of TGFβ on subtyping PDAC CAFs remained unclear until a study demonstrated that the interleukin-1 (IL-1), leukemia inhibitory factor (LIF) and downstream JAK/STAT signaling pathways resulted in the activation of inflammatory CAFs (iCAFs), whereas TGFβ downregulated IL-1R1 to antagonize the activation, and shifted iCAFs to a myofibroblastic phenotype (myoCAFs) in vivo [89]. Based on the fact that most mutations, such as SMAD4 and KRAS, are a downstream signaling factor of TGFβR, and the fact that the mutations of TGFβR accounts for 4–7% of pancreatic cancers [90,91], it can be observed that the formation of PDAC TME is mostly driven by the paracrine or autocrine manner of TGF-β. Besides, it also explains the dual role of TGFβ in the development of PDAC, in which TGFβ exerts primarily antitumor activity on early stage PDAC by means of regulating the cell cycle, apoptosis, and cell differentiation [92], whereas during advanced stages the antitumor response of TGFβ is evaded by cancer cells via the acquisition of mutations in the mediators of the TGFβ pathway [93,94,95,96].

The results of recent preclinical studies have demonstrated therapeutic approaches targeting the TGFβ signaling axis [97,98,99,100]: for example, by silencing the TGFβ2 with Trabedersen (AP 12009), which is an antisense phosphorothioate oligodeoxynucleotide for inhibiting TGFβ2 biosynthesis, Schlingensiepen et al. demonstrated that tumor growth and lymph node metastasis were significantly reduced in a metastatic pancreatic cancer mouse model [101]. The blockade of TGFβ1 by soluble TGFβRII protein also reduced pancreatic cancer cell metastasis in an orthotopic mouse model [97,100]. Other TGF-β inhibitors, such as SD-208, SD-093 and LY2109761, which are inhibitors of TGFβRI/RII kinases, have shown effects in reducing the tumor burden, reducing abdominal metastases, and improving the survival rates for metastatic pancreatic cancer in a murine model [98,102,103,104]. In the clinical setting, Melisi et al. reported that the combination of Galunisertib (LY2157299) with gemcitabine improved overall survival (8.9 months) vs. gemcitabine alone (7.1 months) in patients with unresectable pancreatic cancer (hazard ratio 0.79; 95% CI 0.59 to 1.09) [26]. Losartan, which is an angiotensin II receptor antagonist, was reported to reduce TGFβ signaling and collagen secretion in previous studies [105,106]. More recently in an active phase II trial, Murphy et al. reported that the R0 resection rate of total neoadjuvant FOLFIRINOX (a combination therapy including leucovorin, fluorouracil, irinotecan, and oxaliplatin) and losartan followed by chemoradiotherapy was 61% among all eligible participants, which exceeded expectations in a historically incurable disease associated with prolonged PFS and OS [27].

Given that the CAF and PDAC are mixed together, the therapeutic effects of RTK or TGFβR inhibitors seem to be universal on the cells within PDAC TME. For example, relaxin, a potent inhibitor of TGF-beta, has been demonstrated to inhibit fibroblast differentiation into CAF, impair fibrosis and inhibit tumor growth in PDAC in a pre-clinical study [107]. Although few on-going clinical studies have taken place to date, the findings of preclinical studies provide feasibility that monotherapeutic strategies targeting TGFβR or stroma-targeting therapies in combination with drugs targeting RTK or TGFβR may generate considerably greater tumor responses than monotherapies.

## 4. Conclusions and Future Perspectives

Pancreatic cancer has a potentially disastrous influences on a patient’s life; thus, numerous studies about various RTKs have reformed the knowledge of oncology, leading to the development of novel therapeutic drug candidates. Previously used chemotherapeutic regimens, including FOLFIRINOX or GA (gemcitabine and nab-paclitaxel) present low response rates and high toxicity [108]. Recent results in using inhibitory RTK strategies did not show very exciting efficacy for treating pancreatic cancer patients, but the basis to establish the precision medicine has been provided. Whether the target is expressed in all or some patients and whether this expression correlates with drug responses should be addressed [15]. In addition, the use of cell models and patient-derived xenograft (PDX) animal models, considering role of CAFs and/or TME, may predict the potential efficacy accurately in humans [109]. Detailed downstream signaling of RTKs, such as the KRAS, PI3K-AKT-mTOR and CDC-RAC pathways, tested in cell or animal models illustrating the whole picture may bring solutions, establish different therapeutic combinations or propose new avenues for future research. If these pre-clinically/clinically investigated products translate into the treatments for patients, they will be of the utmost importance, and these drugs would then have a place in the management of pancreatic cancer.

## Figures and Tables

**Table 1:** Representative completed clinical trials assessing the efficacy of RTKs inhibitors on patients with PDAC.

Author/Principal Investigators	NCT Number	Agent	Targets	Treatment	Number of Patients	Phase	Primary Outcome/Objectives	Summary of Results
Moss et al. [15]	NCT0016121	Imatinib mesylate	PDGFR, c-Kit, v-Abl	Drug: Gemcitabine Drug: Imatinib mesylate	44	II	Progression free survival	Imatinib mesylate did not show clinical significance of PFS or OS over GEM monotherapy.
Reni et al. [16]	NCT00967603	Sunitinib malate	PDGFR, FLT3, IRE1α, Kit	No Intervention: Observation Experimental: sunitinib	56	II	Overall survival	The primary end point showing a 6 month-PFS of 22.2% in sunitinib group compared to 3.6% in the calibration arm, while the 2-yr OS did not show significant improvement.
Bergmann et al. [17]	NCT00673504	Sunitinib malate	PDGFR, VEGFR FLT3, IRE1α, Kit	Drug: Gemcitabine and Sunitinib Drug: Gemcitabine	118	II	Time to Progression	Sunitinib malate did not show clinical significance of PFS or OS over GEM monotherapy. However, it was associated with more toxicity.
Moore et al. [18]	NCT00026338	Erlotinib hydrochloride	EGFR	Drug: Erlotinib and Gemcitabine Drug: Gemcitabine	569	III	Overall survival	Erlotinib showed clinical significance of OS over GEM monotherapy with a hazard ratio (HR) of 0.82.
Philip et al. [19]	NCT00075686	Cetuximab	EGFR	Drug: Cetuximab and Gemcitabine Drug: Gemcitabine	766	III	Overall survival	The anti-EGFR monoclonal antibody Cetuximab did not show clinical significance of OS over GEM monotherapy.
Kindler et al. [20]	NCT00088894	Bevacizumab	VEGFR	Drug: Bevacizumab and Gemcitabine Drug: Gemcitabine	590	III	Overall survival	The addition of Bevacizumab did not improve OS over GEM + placebo therapy.
Gonçalves et al. [21]	NCT00541021	Sorafenib	VEGFR	Drug: Sorafenib and Gemcitabine Drug: Gemcitabine	102	III	Progression free survival	The addition of Sorafenib to gemcitabine did not improve PFS.in PDAC patients
Kindler et al. [22]	NCT00471146	Axitinib	VEGFR	Drug: Axitinib and Gemcitabine Drug: Gemcitabine	632	III	Overall survival	The addition of Axitinib to gemcitabine did not improve OS in PDAC patients
Rougier et al. [23]	NCT00574275	Aflibercept	VEGFR	Drug: Aflibercept and Gemcitabine Drug: Gemcitabine	427	III	Overall survival	The addition of Aflibercept to gemcitabine did not improve OS in PDAC patients
Fuchs et al. [24]	NCT01231347	Ganitumab	IGF1R	Drug: Ganitumab and Gemcitabine Drug: Gemcitabine	322	III	Overall survival	The addition of Ganitumab to gemcitabine did not improve OS in PDAC patients
Philip et al. [25]	NCT00617708	Cixutumumab	IGF1R	Drug: Erlotinib, Gemcitabine and Cixutumumab	134	I/II	Progression-Free Survival, Maximum Tolerated Dose Determination	The addition of IGF1R inhibitor, cixutumumab to Erlotinib and G did not improve PFS or OS in metastatic PDAC patients.
Melisi et al. [26]	NCT02734160	Galunisertib	TGFβR	Drug: Galunisertib and Gemcitabine Drug: Gemcitabine	156	I/II	Overall survival	The addition of Galunisertib improved OS and PFS over GEM + placebo therapy.
Murphy et al. [27]	NCT01821729	Losartan	Angiotensin receptor	Drug: FOLFIRINOX, Losartan and proton beam radiation therapy	49	II	Number of participants with R0 resection	The addition of losartan to FOLFIRINOX and chemoradiotherapy downstaged advanced pancreatic ductal adenocarcinoma with an R0 resection rate of 61%.

## Data Availability

Not apllicable.

## Abbreviations

RTK	Receptor tyrosine kinase
TGF	Transforming growth factor beta
CAF	Cancer-associated fibroblast
PDAC	Pancreatic ductal adenocarcinoma
*PDGF*	Platelet-derived growth factor
*PDGFR*	Platelet-derived growth factor receptor
CNS	Central nervous system
MAPK	Mitogen-activated protein kinase
PI3K	Phosphoinositide 3-kinases
ECM	Extracellular matrix
VEGF	Vascular endothelial growth factor
COX-2	Cyclooxygenase-2
NSCLC	Non-small cell lung cancer
OS	Overall survival
PFS	Progression-free survival
IGF1R	Insulinlike growth factor 1 receptor
LIF	Leukemia inhibitory factor
JAK/STAT	Janus kinases (JAKs), signal transducer and activator of transcription proteins (STAT)
SMAD4	Mothers against decapentaplegic homolog 4

## References

[1] Lemmon M.A., Schlessinger J. (2010). Cell signaling by receptor tyrosine kinases. Cell.

[2] Heldin C.H., Moustakas A. (2016). Signaling Receptors for TGF-beta Family Members. Cold Spring Harb. Perspect. Biol..

[3] Du Z., Lovly C.M. (2018). Mechanisms of receptor tyrosine kinase activation in cancer. Mol. Cancer.

[4] Massague J. (2008). TGFbeta in Cancer. Cell.

[5] Takeuchi K., Ito F. (2011). Receptor tyrosine kinases and targeted cancer therapeutics. Biol. Pharm. Bull..

[6] Iqbal N., Iqbal N. (2014). Human Epidermal Growth Factor Receptor 2 (HER2) in Cancers: Overexpression and Therapeutic Implications. Mol. Biol. Int..

[7] Goldstein N.S., Armin M. (2001). Epidermal growth factor receptor immunohistochemical reactivity in patients with American Joint Committee on Cancer Stage IV colon adenocarcinoma: Implications for a standardized scoring system. Cancer.

[8] Donnem T., Al-Saad S., Al-Shibli K., Andersen S., Busund L.T., Bremnes R.M. (2008). Prognostic impact of platelet-derived growth factors in non-small cell lung cancer tumor and stromal cells. J. Thorac. Oncol..

[9] Ozdemir F., Akdogan R., Aydin F., Reis A., Kavgaci H., Gul S., Akdogan E. (2006). The effects of VEGF and VEGFR-2 on survival in patients with gastric cancer. J. Exp. Clin. Cancer. Res..

[10] Arcaro A. (2013). Targeting the insulin-like growth factor-1 receptor in human cancer. Front. Pharmacol..

[11] Papageorgis P., Stylianopoulos T. (2015). Role of TGFbeta in regulation of the tumor microenvironment and drug delivery (review). Int. J. Oncol..

[12] Reck M., van Zandwijk N., Gridelli C., Baliko Z., Rischin D., Allan S., Krzakowski M., Heigener D. (2010). Erlotinib in Advanced Non-small Cell Lung Cancer: Efficacy and Safety Findings of the Global Phase IV Tarceva Lung Cancer Survival Treatment Study. J. Thorac. Oncol..

[13] Dhillon S. (2018). Regorafenib: A Review in Metastatic Colorectal Cancer. Drugs.

[14] Pandol S., Edderkaoui M., Gukovsky I., Lugea A., Gukovskaya A. (2009). Desmoplasia of pancreatic ductal adenocarcinoma. Clin. Gastroenterol. Hepatol..

[15] Moss R.A., Moore D., Mulcahy M.F., Nahum K., Saraiya B., Eddy S., Kleber M., Poplin E.A. (2012). A Multi-institutional Phase 2 Study of Imatinib Mesylate and Gemcitabine for First-Line Treatment of Advanced Pancreatic Cancer. Gastrointest. Cancer Res..

[16] Reni M., Cereda S., Milella M., Novarino A., Passardi A., Mambrini A., Di Lucca G., Aprile G., Belli C., Danova M. (2013). Maintenance sunitinib or observation in metastatic pancreatic adenocarcinoma: A phase II randomised trial. Eur. J. Cancer.

[17] Bergmann L., Maute L., Heil G., Rüssel J., Weidmann E., Köberle D., Fuxius S., Weigang-Köhler K., Aulitzky W.E., Wörmann B. (2015). A prospective randomised phase-II trial with gemcitabine versus gemcitabine plus sunitinib in advanced pancreatic cancer: A study of the CESAR Central European Society for Anticancer Drug Research-EWIV. Eur. J. Cancer.

[18] Moore M.J., Goldstein D., Hamm J., Figer A., Hecht J.R., Gallinger S., Au H.J., Murawa P., Walde D., Wolff R.A. (2007). Erlotinib plus gemcitabine compared with gemcitabine alone in patients with advanced pancreatic cancer: A phase III trial of the National Cancer Institute of Canada Clinical Trials Group. J. Clin. Oncol..

[19] Philip P.A., Benedetti J., Corless C.L., Wong R., O’Reilly E.M., Flynn P.J., Rowland K.M., Atkins J.N., Mirtsching B.C., Rivkin S.E. (2010). Phase III study comparing gemcitabine plus cetuximab versus gemcitabine in patients with advanced pancreatic adenocarcinoma: Southwest Oncology Group-directed intergroup trial S0205. J. Clin. Oncol..

[20] Kindler H.L., Niedzwiecki D., Hollis D., Sutherland S., Schrag D., Hurwitz H., Innocenti F., Mulcahy M.F., O’Reilly E., Wozniak T.F. (2010). Gemcitabine plus bevacizumab compared with gemcitabine plus placebo in patients with advanced pancreatic cancer: Phase III trial of the Cancer and Leukemia Group B (CALGB 80303). J. Clin. Oncol..

[21] Gonçalves A., Gilabert M., François E., Dahan L., Perrier H., Lamy R., Re D., Largillier R., Gasmi M., Tchiknavorian X. (2012). BAYPAN study: A double-blind phase III randomized trial comparing gemcitabine plus sorafenib and gemcitabine plus placebo in patients with advanced pancreatic cancer. Ann. Oncol..

[22] Kindler H.L., Ioka T., Richel D.J., Bennouna J., Letourneau R., Okusaka T., Funakoshi A., Furuse J., Park Y.S., Ohkawa S. (2011). Axitinib plus gemcitabine versus placebo plus gemcitabine in patients with advanced pancreatic adenocarcinoma: A double-blind randomised phase 3 study. Lancet Oncol..

[23] Rougier P., Riess H., Manges R., Karasek P., Humblet Y., Barone C., Santoro A., Assadourian S., Hatteville L., Philip P.A. (2013). Randomised, placebo-controlled, double-blind, parallel-group phase III study evaluating aflibercept in patients receiving first-line treatment with gemcitabine for metastatic pancreatic cancer. Eur. J. Cancer.

[24] Fuchs C.S., Azevedo S., Okusaka T., Van Laethem J.L., Lipton L.R., Riess H., Szczylik C., Moore M.J., Peeters M., Bodoky G. (2015). A phase 3 randomized, double-blind, placebo-controlled trial of ganitumab or placebo in combination with gemcitabine as first-line therapy for metastatic adenocarcinoma of the pancreas: The GAMMA trial. Ann. Oncol..

[25] Philip P.A., Goldman B., Ramanathan R.K., Lenz H.J., Lowy A.M., Whitehead R.P., Wakatsuki T., Iqbal S., Gaur R., Benedetti J.K. (2014). Dual blockade of epidermal growth factor receptor and insulin-like growth factor receptor-1 signaling in metastatic pancreatic cancer: Phase Ib and randomized phase II trial of gemcitabine, erlotinib, and cixutumumab versus gemcitabine plus erlotinib (SWOG S0727). Cancer.

[26] Melisi D., Garcia-Carbonero R., Macarulla T., Pezet D., Deplanque G., Fuchs M., Trojan J., Oettle H., Kozloff M., Cleverly A. (2018). Galunisertib plus gemcitabine vs. gemcitabine for first-line treatment of patients with unresectable pancreatic cancer. Br. J. Cancer.

[27] Murphy J.E., Wo J.Y., Ryan D.P., Clark J.W., Jiang W., Yeap B.Y., Drapek L.C., Ly L., Baglini C.V., Blaszkowsky L.S. (2019). Total Neoadjuvant Therapy with FOLFIRINOX in Combination with Losartan Followed by Chemoradiotherapy for Locally Advanced Pancreatic Cancer: A Phase 2 Clinical Trial. JAMA Oncol..

[28] Betsholtz C. (2004). Insight into the physiological functions of PDGF through genetic studies in mice. Cytokine Growth Factor Rev..

[29] Östman A., Heldin C.H. (2007). PDGF Receptors as Targets in Tumor Treatment. Advances in Cancer Research.

[30] Karagiannis G.S., Poutahidis T., Erdman S.E., Kirsch R., Riddell R.H., Diamandis E.P. (2012). Cancer-associated fibroblasts drive the progression of metastasis through both paracrine and mechanical pressure on cancer tissue. Mol. Cancer Res..

[31] Pickup M.W., Mouw J.K., Weaver V.M. (2014). The extracellular matrix modulates the hallmarks of cancer. EMBO Rep..

[32] Shao Z.M., Nguyen M., Barsky S.H. (2000). Human breast carcinoma desmoplasia is PDGF initiated. Oncogene.

[33] Tejada M.L., Yu L., Dong J., Jung K., Meng G., Peale F.V., Frantz G.D., Hall L., Liang X., Gerber H.P. (2006). Tumor-driven paracrine platelet-derived growth factor receptor alpha signaling is a key determinant of stromal cell recruitment in a model of human lung carcinoma. Clin. Cancer Res..

[34] Bandapalli O.R., Macher-Goeppinger S., Schirmacher P., Brand K. (2012). Paracrine signalling in colorectal liver metastases involving tumor cell-derived PDGF-C and hepatic stellate cell-derived PAK-2. Clin. Exp. Metastasis.

[35] Cullen K.J., Smith H.S., Hill S., Rosen N., Lippman M.E. (1991). Growth factor messenger RNA expression by human breast fibroblasts from benign and malignant lesions. Cancer Res..

[36] Bartoschek M., Oskolkov N., Bocci M., Lovrot J., Larsson C., Sommarin M., Madsen C.D., Lindgren D., Pekar G., Karlsson G. (2018). Spatially and functionally distinct subclasses of breast cancer-associated fibroblasts revealed by single cell RNA sequencing. Nat. Commun..

[37] Costa A., Kieffer Y., Scholer-Dahirel A., Pelon F., Bourachot B., Cardon M., Sirven P., Magagna I., Fuhrmann L., Bernard C. (2018). Fibroblast Heterogeneity and Immunosuppressive Environment in Human Breast Cancer. Cancer Cell.

[38] Seymour L., Dajee D., Bezwoda W.R. (1993). Tissue platelet derived-growth factor (PDGF) predicts for shortened survival and treatment failure in advanced breast cancer. Breast Cancer Res. Treat..

[39] Yuzawa S., Kano M.R., Einama T., Nishihara H. (2012). PDGFRbeta expression in tumor stroma of pancreatic adenocarcinoma as a reliable prognostic marker. Med. Oncol..

[40] Fjallskog M.L., Hessman O., Eriksson B., Janson E.T. (2007). Upregulated expression of PDGF receptor beta in endocrine pancreatic tumors and metastases compared to normal endocrine pancreas. Acta Oncol..

[41] Ebert M., Yokoyama M., Friess H., Kobrin M.S., Buchler M.W., Korc M. (1995). Induction of platelet-derived growth factor A and B chains and over-expression of their receptors in human pancreatic cancer. Int. J. Cancer.

[42] Ohlund D., Handly-Santana A., Biffi G., Elyada E., Almeida A.S., Ponz-Sarvise M., Corbo V., Oni T.E., Hearn S.A., Lee E.J. (2017). Distinct populations of inflammatory fibroblasts and myofibroblasts in pancreatic cancer. J. Exp. Med..

[43] Neuzillet C., Tijeras-Raballand A., Ragulan C., Cros J., Patil Y., Martinet M., Erkan M., Kleeff J., Wilson J., Apte M. (2019). Inter- and intra-tumoural heterogeneity in cancer-associated fibroblasts of human pancreatic ductal adenocarcinoma. J. Pathol..

[44] Kantarjian H., Sawyers C., Hochhaus A., Guilhot F., Schiffer C., Gambacorti-Passerini C., Niederwieser D., Resta D., Capdeville R., Zoellner U. (2002). Hematologic and cytogenetic responses to imatinib mesylate in chronic myelogenous leukemia. N. Engl. J. Med..

[45] Goodman V.L., Rock E.P., Dagher R., Ramchandani R.P., Abraham S., Gobburu J.V., Booth B.P., Verbois S.L., Morse D.E., Liang C.Y. (2007). Approval summary: Sunitinib for the treatment of imatinib refractory or intolerant gastrointestinal stromal tumors and advanced renal cell carcinoma. Clin. Cancer Res..

[46] Llovet J.M., Ricci S., Mazzaferro V., Hilgard P., Gane E., Blanc J.F., de Oliveira A.C., Santoro A., Raoul J.L., Forner A. (2008). Sorafenib in advanced hepatocellular carcinoma. N. Engl. J. Med..

[47] Kindler H.L., Wroblewski K., Wallace J.A., Hall M.J., Locker G., Nattam S., Agamah E., Stadler W.M., Vokes E.E. (2012). Gemcitabine plus sorafenib in patients with advanced pancreatic cancer: A phase II trial of the University of Chicago Phase II Consortium. Investig. New Drugs.

[48] Pines G., Kostler W.J., Yarden Y. (2010). Oncogenic mutant forms of EGFR: Lessons in signal transduction and targets for cancer therapy. FEBS Lett..

[49] Fisher D.A., Lakshmanan J. (1990). Metabolism and effects of epidermal growth factor and related growth factors in mammals. Endocr. Rev..

[50] Chia C.M., Winston R.M., Handyside A.H. (1995). EGF, TGF-alpha and EGFR expression in human preimplantation embryos. Development.

[51] Sigismund S., Avanzato D., Lanzetti L. (2018). Emerging functions of the EGFR in cancer. Mol. Oncol..

[52] Levental K.R., Yu H., Kass L., Lakins J.N., Egeblad M., Erler J.T., Fong S.F., Csiszar K., Giaccia A., Weninger W. (2009). Matrix crosslinking forces tumor progression by enhancing integrin signaling. Cell.

[53] Grasset E.M., Bertero T., Bozec A., Friard J., Bourget I., Pisano S., Lecacheur M., Maiel M., Bailleux C., Emelyanov A. (2018). Matrix Stiffening and EGFR Cooperate to Promote the Collective Invasion of Cancer Cells. Cancer Res..

[54] Yarwood S.J., Woodgett J.R. (2001). Extracellular matrix composition determines the transcriptional response to epidermal growth factor receptor activation. Proc. Natl. Acad. Sci. USA.

[55] Hu H., Han T., Zhuo M., Wu L.L., Yuan C., Wu L., Lei W., Jiao F., Wang L.W. (2017). Elevated COX-2 Expression Promotes Angiogenesis Through EGFR/p38-MAPK/Sp1-Dependent Signalling in Pancreatic Cancer. Sci. Rep..

[56] Ma X., Wu D., Zhou S., Wan F., Liu H., Xu X., Xu X., Zhao Y., Tang M. (2016). The pancreatic cancer secreted REG4 promotes macrophage polarization to M2 through EGFR/AKT/CREB pathway. Oncol. Rep..

[57] Ciardiello F., Tortora G. (2008). EGFR antagonists in cancer treatment. N. Engl. J. Med..

[58] Cheng L., Alexander R.E., Maclennan G.T., Cummings O.W., Montironi R., Lopez-Beltran A., Cramer H.M., Davidson D.D., Zhang S. (2012). Molecular pathology of lung cancer: Key to personalized medicine. Mod. Pathol..

[59] Rusch V., Klimstra D., Venkatraman E., Pisters P.W., Langenfeld J., Dmitrovsky E. (1997). Overexpression of the epidermal growth factor receptor and its ligand transforming growth factor alpha is frequent in resectable non-small cell lung cancer but does not predict tumor progression. Clin. Cancer Res..

[60] Korc M., Chandrasekar B., Yamanaka Y., Friess H., Buchier M., Beger H.G. (1992). Overexpression of the epidermal growth factor receptor in human pancreatic cancer is associated with concomitant increases in the levels of epidermal growth factor and transforming growth factor alpha. J. Clin. Investig..

[61] Wang J.P., Wu C.-Y., Yeh Y.-C., Shyr Y.-M., Wu Y.-Y., Kuo C.-Y., Hung Y.-P., Chen M.-H., Lee W.-P., Luo J.-C. (2015). Erlotinib is effective in pancreatic cancer with epidermal growth factor receptor mutations: A randomized, open-label, prospective trial. Oncotarget.

[62] Oliveira-Cunha M., Hadfield K.D., Siriwardena A.K., Newman W. (2012). EGFR and KRAS mutational analysis and their correlation to survival in pancreatic and periampullary cancer. Pancreas.

[63] Navas C., Hernández-Porras I., Schuhmacher A.J., Sibilia M., Guerra C., Barbacid M. (2012). EGF receptor signaling is essential for k-ras oncogene-driven pancreatic ductal adenocarcinoma. Cancer Cell.

[64] Imai K., Takaoka A. (2006). Comparing antibody and small-molecule therapies for cancer. Nat. Rev. Cancer.

[65] Conradt L., Godl K., Schaab C., Tebbe A., Eser S., Diersch S., Michalski C.W., Kleeff J., Schnieke A., Schmid R.M. (2011). Disclosure of erlotinib as a multikinase inhibitor in pancreatic ductal adenocarcinoma. Neoplasia.

[66] Kim D., Xue J.Y., Lito P. (2020). Targeting KRAS(G12C): From Inhibitory Mechanism to Modulation of Antitumor Effects in Patients. Cell.

[67] Seo Y., Baba H., Fukuda T., Takashima M., Sugimachi K. (2000). High expression of vascular endothelial growth factor is associated with liver metastasis and a poor prognosis for patients with ductal pancreatic adenocarcinoma. Cancer.

[68] Astsaturov I.A., Meropol N.J., Alpaugh R.K., Burtness B.A., Cheng J.D., McLaughlin S., Rogatko A., Xu Z., Watson J.C., Weiner L.M. (2011). Phase II and coagulation cascade biomarker study of bevacizumab with or without docetaxel in patients with previously treated metastatic pancreatic adenocarcinoma. Am. J. Clin. Oncol..

[69] Van Cutsem E., Vervenne W.L., Bennouna J., Humblet Y., Gill S., Van Laethem J.L., Verslype C., Scheithauer W., Shang A., Cosaert J. (2009). Phase III trial of bevacizumab in combination with gemcitabine and erlotinib in patients with metastatic pancreatic cancer. J. Clin. Oncol..

[70] Wit J.M., Walenkamp M.J. (2013). Role of insulin-like growth factors in growth, development and feeding. World Rev. Nutr. Diet.

[71] Yakar S., Kim H., Zhao H., Toyoshima Y., Pennisi P., Gavrilova O., Leroith D. (2005). The growth hormone-insulin like growth factor axis revisited: Lessons from IGF-1 and IGF-1 receptor gene targeting. Pediatr. Nephrol..

[72] Russo V.C., Gluckman P.D., Feldman E.L., Werther G.A. (2005). The insulin-like growth factor system and its pleiotropic functions in brain. Endocr. Rev..

[73] Moody G., Beltran P.J., Mitchell P., Cajulis E., Chung Y.A., Hwang D., Kendall R., Radinsky R., Cohen P., Calzone F.J. (2014). IGF1R blockade with ganitumab results in systemic effects on the GH-IGF axis in mice. J. Endocrinol..

[74] Delafontaine P., Song Y.H., Li Y. (2004). Expression, regulation, and function of IGF-1, IGF-1R, and IGF-1 binding proteins in blood vessels. Arterioscler. Thromb. Vasc. Biol..

[75] Gao S., Wassler M., Zhang L., Li Y., Wang J., Zhang Y., Shelat H., Williams J., Geng Y.J. (2014). MicroRNA-133a regulates insulin-like growth factor-1 receptor expression and vascular smooth muscle cell proliferation in murine atherosclerosis. Atherosclerosis.

[76] Valsecchi M.E., McDonald M., Brody J.R., Hyslop T., Freydin B., Yeo C.J., Solomides C., Peiper S.C., Witkiewicz A.K. (2012). Epidermal growth factor receptor and insulinlike growth factor 1 receptor expression predict poor survival in pancreatic ductal adenocarcinoma. Cancer.

[77] Peretz S., Kim C., Rockwell S., Baserga R., Glazer P.M. (2002). IGF1 receptor expression protects against microenvironmental stress found in the solid tumor. Radiat. Res..

[78] Tape C.J., Ling S., Dimitriadi M., McMahon K.M., Worboys J.D., Leong H.S., Norrie I.C., Miller C.J., Poulogiannis G., Lauffenburger D.A. (2016). Oncogenic KRAS Regulates Tumor Cell Signaling via Stromal Reciprocation. Cell.

[79] Rucki A.A., Foley K., Zhang P., Xiao Q., Kleponis J., Wu A.A., Sharma R., Mo G., Liu A., Van Eyk J. (2017). Heterogeneous Stromal Signaling within the Tumor Microenvironment Controls the Metastasis of Pancreatic Cancer. Cancer Res..

[80] Scales S.J., de Sauvage F.J. (2009). Mechanisms of Hedgehog pathway activation in cancer and implications for therapy. Trends Pharmacol. Sci..

[81] Seoane J. (2006). Escaping from the TGFbeta anti-proliferative control. Carcinogenesis.

[82] Principe D.R., Doll J.A., Bauer J., Jung B., Munshi H.G., Bartholin L., Pasche B., Lee C., Grippo P.J. (2014). TGF-beta: Duality of function between tumor prevention and carcinogenesis. J. Natl. Cancer Inst..

[83] Seoane J., Le H.V., Shen L., Anderson S.A., Massague J. (2004). Integration of Smad and forkhead pathways in the control of neuroepithelial and glioblastoma cell proliferation. Cell.

[84] Papageorgis P. (2015). TGFbeta Signaling in Tumor Initiation, Epithelial-to-Mesenchymal Transition, and Metastasis. J. Oncol..

[85] Padua D., Massague J. (2009). Roles of TGFbeta in metastasis. Cell Res..

[86] Friess H., Yamanaka Y., Buchler M., Ebert M., Beger H.G., Gold L.I., Korc M. (1993). Enhanced expression of transforming growth factor beta isoforms in pancreatic cancer correlates with decreased survival. Gastroenterology.

[87] Wagner M., Kleeff J., Friess H., Buchler M.W., Korc M. (1999). Enhanced expression of the type II transforming growth factor-beta receptor is associated with decreased survival in human pancreatic cancer. Pancreas.

[88] Javle M., Li Y., Tan D., Dong X., Chang P., Kar S., Li D. (2014). Biomarkers of TGF-beta signaling pathway and prognosis of pancreatic cancer. PLoS ONE.

[89] Biffi G., Oni T.E., Spielman B., Hao Y., Elyada E., Park Y., Preall J., Tuveson D.A. (2019). IL1-Induced JAK/STAT Signaling Is Antagonized by TGFbeta to Shape CAF Heterogeneity in Pancreatic Ductal Adenocarcinoma. Cancer Discov..

[90] Lin X., Feng X.H. (2005). Abrogation of transforming growth factor-beta signaling in pancreatic cancer. World J. Surg..

[91] Hansel D.E., Kern S.E., Hruban R.H. (2003). Molecular pathogenesis of pancreatic cancer. Annu. Rev. Genom. Hum. Genet..

[92] Kubiczkova L., Sedlarikova L., Hajek R., Sevcikova S. (2012). TGF-beta—An excellent servant but a bad master. J. Transl. Med..

[93] Markowitz S., Wang J., Myeroff L., Parsons R., Sun L., Lutterbaugh J., Fan R.S., Zborowska E., Kinzler K.W., Vogelstein B. (1995). Inactivation of the type II TGF-beta receptor in colon cancer cells with microsatellite instability. Science.

[94] Zhao M., Mishra L., Deng C.X. (2018). The role of TGF-beta/SMAD4 signaling in cancer. Int. J. Biol. Sci..

[95] Bardeesy N., Cheng K.H., Berger J.H., Chu G.C., Pahler J., Olson P., Hezel A.F., Horner J., Lauwers G.Y., Hanahan D. (2006). Smad4 is dispensable for normal pancreas development yet critical in progression and tumor biology of pancreas cancer. Genes Dev..

[96] Wang F., Xia X., Yang C., Shen J., Mai J., Kim H.C., Kirui D., Kang Y., Fleming J.B., Koay E.J. (2018). SMAD4 Gene Mutation Renders Pancreatic Cancer Resistance to Radiotherapy through Promotion of Autophagy. Clin. Cancer Res..

[97] Rowland-Goldsmith M.A., Maruyama H., Matsuda K., Idezawa T., Ralli M., Ralli S., Korc M. (2002). Soluble type II transforming growth factor-beta receptor attenuates expression of metastasis-associated genes and suppresses pancreatic cancer cell metastasis. Mol. Cancer Ther..

[98] Melisi D., Ishiyama S., Sclabas G.M., Fleming J.B., Xia Q., Tortora G., Abbruzzese J.L., Chiao P.J. (2008). LY2109761, a novel transforming growth factor beta receptor type I and type II dual inhibitor, as a therapeutic approach to suppressing pancreatic cancer metastasis. Mol. Cancer Ther..

[99] Arteaga C.L. (2006). Inhibition of TGFbeta signaling in cancer therapy. Curr. Opin. Genet. Dev..

[100] Rowland-Goldsmith M.A., Maruyama H., Kusama T., Ralli S., Korc M. (2001). Soluble type II transforming growth factor-beta (TGF-beta) receptor inhibits TGF-beta signaling in COLO-357 pancreatic cancer cells in vitro and attenuates tumor formation. Clin. Cancer Res..

[101] Schlingensiepen K.H., Jaschinski F., Lang S.A., Moser C., Geissler E.K., Schlitt H.J., Kielmanowicz M., Schneider A. (2011). Transforming growth factor-beta 2 gene silencing with trabedersen (AP 12009) in pancreatic cancer. Cancer Sci..

[102] Gaspar N.J., Li L., Kapoun A.M., Medicherla S., Reddy M., Li G., O’Young G., Quon D., Henson M., Damm D.L. (2007). Inhibition of transforming growth factor beta signaling reduces pancreatic adenocarcinoma growth and invasiveness. Mol. Pharmacol..

[103] Medicherla S., Li L., Ma J.Y., Kapoun A.M., Gaspar N.J., Liu Y.W., Mangadu R., O’Young G., Protter A.A., Schreiner G.F. (2007). Antitumor activity of TGF-beta inhibitor is dependent on the microenvironment. Anticancer Res..

[104] Subramanian G., Schwarz R.E., Higgins L., McEnroe G., Chakravarty S., Dugar S., Reiss M. (2004). Targeting endogenous transforming growth factor beta receptor signaling in SMAD4-deficient human pancreatic carcinoma cells inhibits their invasive phenotype1. Cancer Res..

[105] Nataatmadja M., West J., Prabowo S., West M. (2013). Angiotensin II Receptor Antagonism Reduces Transforming Growth Factor Beta and Smad Signaling in Thoracic Aortic Aneurysm. Ochsner J..

[106] Campistol J.M., Inigo P., Jimenez W., Lario S., Clesca P.H., Oppenheimer F., Rivera F. (1999). Losartan decreases plasma levels of TGF-beta1 in transplant patients with chronic allograft nephropathy. Kidney Int..

[107] Mardhian D.F., Storm G., Bansal R., Prakash J. (2018). Nano-targeted relaxin impairs fibrosis and tumor growth in pancreatic cancer and improves the efficacy of gemcitabine in vivo. J. Control. Release.

[108] Tempero M.A., Malafa M.P., Al-Hawary M., Behrman S.W., Benson A.B., Cardin D.B., Chiorean E.G., Chung V., Czito B., Del Chiaro M. (2021). Pancreatic Adenocarcinoma, Version 2.2021, NCCN Clinical Practice Guidelines in Oncology. J. Natl. Compr. Cancer Netw..

[109] Xu Z., Pang T.C.Y., Liu A.C., Pothula S.P., Mekapogu A.R., Perera C.J., Murakami T., Goldstein D., Pirola R.C., Wilson J.S. (2020). Targeting the HGF/c-MET pathway in advanced pancreatic cancer: A key element of treatment that limits primary tumour growth and eliminates metastasis. Br. J. Cancer.

